# Thermal and Photochemical Reactions of Organosilicon Compounds

**DOI:** 10.3390/molecules30051158

**Published:** 2025-03-04

**Authors:** Masae Takahashi

**Affiliations:** Department of Physics, Graduate School of Science, Tohoku University, Sendai 980-8578, Japan; masae.takahashi.d1@tohoku.ac.jp

**Keywords:** computational chemistry, reaction process, organosilicon compound

## Abstract

This article provides a comprehensive review of quantum chemical computational studies on the thermal and photochemical reactions of organosilicon compounds, based on fundamental concepts such as initial complex formation, HOMO-LUMO interactions, and subjacent orbital interactions. Despite silicon’s position in group 14 of the periodic table, alongside carbon, its reactivity patterns exhibit significant deviations from those of carbon. This review delves into the reactivity behaviors of organosilicon compounds, particularly focusing on the highly coordinated nature of silicon. It is poised to serve as a valuable resource for chemists, offering insights into cutting-edge research and fostering further innovations in synthetic chemistry and also theoretical chemistry.

## 1. Introduction

Ab initio molecular orbital (MO) calculations are a powerful tool for investigating reaction pathways from reactants to products, offering insights for controlling these pathways to obtain desired molecules. This review introduces the theoretical process of identifying reaction pathways based on chemical intuition, prior to the advent of automated programs designed to determine reaction pathways. This approach is fascinating and even informative from a chemical perspective.

Silicon, a group 14 element, shares chemical properties with carbon. However, while these similarities hold true in some respects, they do not extend to the structure and reactivity. This review covers thermal and photochemical reactions of organosilicon compounds. In the thermal reaction section, bimolecular 1,2-addition reactions to double bonds and unimolecular 1,3-rearrangements are discussed. The photochemical reaction section addresses interconversion and unimolecular sigmatropic rearrangements. The treatment of excited states, which differ significantly from ground state phenomena, includes a detailed explanation of the key process of selecting the complete active space.

## 2. Thermal Reactions

### 2.1. 1,2-Additions to the Double Bonds

The differences in stable structures between unsaturated silicon and carbon compounds result in varied reactivity and selectivity of chemical reactions. Unsaturated carbon compounds, such as ethylene, acetylene, allene, benzene, and the two-dimensional material graphene, are stable, exhibiting planar or linear structures. Conversely, their silicon counterparts, including disilene, disilyne, trisilaallene, hexasilabenzene, and silicene, are unstable, likely due to their nonplanar or nonlinear structures. Silicene, the silicon equivalent of graphene, has been experimentally synthesized in a vacuum as a promising material and theoretically studied [1,2,3,4,5,6,7,8,9,10,11,12,13]. However, a significant issue is that silicene sheets oxidize and decompose in air due to their buckled structure [14]. The instability of unsaturated silicon compounds has necessitated considerable experimental efforts for their isolation. Disilene, disilyne, and trisilaallene were ultimately isolated using bulky substituents [15,16,17,18]. The experimentally isolated structures of these compounds adopt nonplanar or nonlinear configurations. A notable feature of the molecular structures of four-membered cyclic silenes in the solid state, as determined by single-crystal X-ray diffraction, is the prominent pyramidal silicon atom [19]. Sterically protected Si=P double bonds have been generated and structurally characterized [20,21,22,23,24]. Additionally, compounds such as phosphaallenes [25,26,27,28,29,30,31,32,33,34], silaallenes [35,36,37,38], germasilaallenes [39,40], and 1-phospha-3-silaallene [41] have been synthesized and studied theoretically [42,43,44,45,46,47,48,49]. Theoretical studies have highlighted that traditional density functionals like B3LYP or BP86 are inadequate for properly describing these bulky molecular structures [50]. Unfortunately, the air-stable planar hexasilabenzene and silicene have not yet been achieved experimentally. While steric stabilization using bulky substituents is common in experiments, several planar or linear unsaturated silicon compounds, expected to be air-stable, have been theoretically proposed through electronic stabilization [51,52,53,54,55,56,57,58,59].

**Figure 1:**
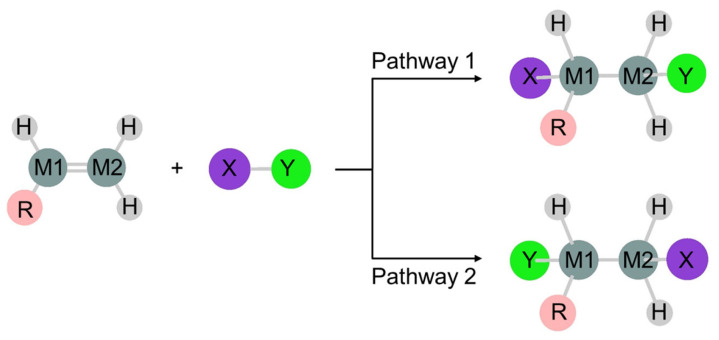
1,2-Addition reaction of molecule XY to doubly bonded compound RHM1=M2H_2_, resulting in two regioselective products RHXM1–M2YH_2_ and RHYM1–M2XH_2_ via pathways 1 and 2, respectively. For water addition to disilene, X, Y, M1, and M2 are H, OH, Si, and Si, respectively.

In the 1,2-addition of the molecule XY to the doubly bonded compound >M1=M2<, the two reactant molecules follow two pathways 1 and 2, resulting in two regioselective products (Figure 1). Disilene, a silicon–silicon double-bonded compound, reacts readily with water, alcohol, and haloalkane to form the corresponding adducts [60,61,62,63,64,65,66,67,68,69]. This type of reaction does not occur easily with olefin, a carbon–carbon double-bonded compound, making the reaction mechanism of 1,2-additions to disilene a topic of significant interest for organic chemists.

#### 2.1.1. The Formation of Initial Complexes

In theoretical studies of reaction pathways, the transition state of the reaction is of primary interest, as it is key to determining the product. However, the initial stage, where the two molecules encounter and the reaction begins, has received less attention. Discovering an initial complex, where the two reactants are weakly bonded, is crucial for comprehensively exploring the reaction pathway, including those not evident through common sense. In some reactions of silenes, silicon–carbon double-bonded compounds, the formation of initial complexes has been experimentally suggested. Wiberg proposed a two-step mechanism, involving the formation of an initial silene–alcohol complex, followed by proton migration from the alcohol to the carbon of the silene [70,71]. A kinetic study of the addition of acetone to silatriene indicated a stepwise pathway involving an initial attack by the carbonyl oxygen on the silenic silicon atom [72]. Kinetic studies of the ene-addition of acetone to diphenylsilene support a stepwise mechanism involving the rapid and reversible formation of a zwitterionic silene–ketone complex, followed by a rate-limiting proton transfer from the ketone to the silene [73,74]. Based on substituent, isotope, and temperature effects on the rate constants for addition reactions to 1,1-diphenylsilene, it is concluded that these reactions proceed via the following stepwise mechanism: the nucleophilic site of the reagent first attacks silicon reversibly to generate an intermediate, which then proceeds to the product in a subsequent electrophile transfer step [73,75,76]. Takahashi et al. theoretically revealed the existence of an initial complex at the start of the 1,2-addition reaction of water to disilene, where two reactant molecules are in weak contact through van der Waals (VDW) interactions [77].

Disilenes react very smoothly with water and various alcohols without a catalyst, forming the corresponding adducts [60,61,62,63,64,65,66,67,68,69]. However, the origin of the diverse stereochemical outcomes has been controversial. The reaction of the stereoisomeric disilene, (*E*)-1,2-di-*tert*-butyl-1,2-dimesityldisilene, with various alcohols resulted in a mixture of two diastereomers [78]. The diastereoselectivity of the reaction of transient disilenes (*E*)- and (*Z*)-PhMeSi=SiMePh with alcohols was controlled by the alcohol concentration [79], suggesting intra- and intermolecular migrations of the alcoholic hydrogen atom. The addition of *p*-methoxyphenol to (*E*)-1,2-di-*tert*-butyl-1,2-dimesityldisilene was shown to be *syn*-selective in benzene but *anti*-selective in polar THF, even at low alcohol concentrations [80], indicating a bimolecular reaction of disilene with alcohols to an *anti*-adduct. The stereochemical diversity has been explained by the competition between the rotation around the Si–Si bond of the zwitterionic intermediate and the intramolecular proton transfer, but no clear explanation has been given for significant solvent effects. Takahashi et al. used ab initio MO calculations to reveal the formation of two VDW complexes at the initial stage of the reaction of disilene with water, elucidating the origin of the remarkable stereochemical diversity [77].

Predicting the reaction pathways is an intriguing topic for theoretical chemists. Frontier molecular orbital (FMO) theory is well known for predicting the qualitative characteristics of many organic reactions, including their reactivity, substituent effects, and stereoselectivity [81,82]. Since this theory considers the HOMO and LUMO of the reacting molecules, it is only applicable to the initial stages of the reaction and is less useful for multistep reactions. Modern theoretical approaches to reaction mechanisms involve searching for transition states through ab initio MO calculations and then finding paths from the transition states along the intrinsic reaction coordinates.

**Figure 2:**
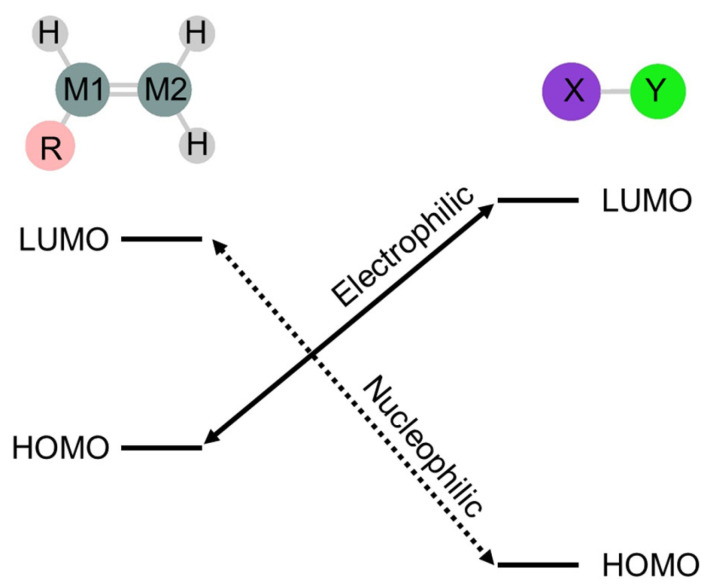
HOMO–LUMO interaction between molecule XY and doubly bonded compound RHM1=M2H_2_.

FMO theory is effective for the initial stage of chemical reactions [77,81,82]. In the 1,2-addition of molecule XY to doubly bonded compound RHM1=M2H_2_, two sets of initial interactions are predicted: nucleophilic attack by the lowest unoccupied molecular orbital (LUMO) of XY on the highest occupied molecular orbital (HOMO) of RHM1=M2H_2_, and electrophilic attack by the HOMO of XY on the LUMO of RHM1=M2H_2_ (Figure 2). Both interactions generate weakly coupled initial complexes, the nucleophilic initial complex C_N_ and the electrophilic complex C_E_. The existence of these complexes depends on the relative energy levels of the interacting orbitals.

Reactions **R1**–**R4** begin with the formation of two types of initial complexes, C_N_ and C_E_. Water and methanol act as both nucleophiles and electrophiles [77,83].

**R1** [77]: H_2_Si=SiH_2_ + H_2_O
**R2** [83]: H_2_Si=SiH_2_ + CH_3_OH
**R3** [83]: H_2_Si=CH_2_ + H_2_O
**R4** [83]: H_2_Si=CH_2_ + CH_3_OH

One key finding in **R1** is the bimolecular *anti*-addition pathway from C_N_ [77]. The existence of the *anti*-addition pathway has also been suggested experimentally [80]. Theoretical findings [77] and experimental results [80] suggest a different mechanism than previously believed. The existence of the two initial complexes C_N_ and C_E_ and the characteristic reaction channel in **R2** are similar to those in **R1** [83]. The Si=C bond in the reactants of reactions **R3** and **R4** is strongly polarized, due to the large difference in electronegativity between carbon and silicon, making the silicon in this bond more positive than that in disilene [83]. Thus, only the C_E_ complex is expected for the attack on carbon, and only the C_N_ complex is expected for the attack on silicon.

In reactions **R5**–**R13**, the C_N_ type of initial complexes is missing [83,84].

**R5** [83]: H_2_Si=SiH_2_ + CF_3_OH
**R6** [83]: H_2_C=CH_2_ + H_2_O
**R7** [83]: H_2_C=CH_2_ + CH_3_OH
**R8** [84]: H_2_C=CH_2_ + HF
**R9** [84]: H_2_C=CH_2_ + HCl
**R10** [84]: H_2_Si=SiH_2_ + HF
**R11** [84]: H_2_Si=SiH_2_ + HCl
**R12** [84]: H_2_Si=CH_2_ + HF
**R13** [84]: H_2_Si=CH_2_ + HCl

The OH group of trifluoromethanol is less electronegative than that of water and methanol, so reaction **R5** provides only C_E_-type complexes [83]. Because carbon is more electronegative than silicon, only electrophilic complexes C_E_ are formed in reactions **R6** and **R7** [83]. In reactions **R8**–**R13**, the nucleophilic complexes C_N_ are not obtained due to the strong acidity of the hydrogen in HY (Y = F, Cl) [84].

The initial complexes involved in the 1,2-addition to monosubstituted disilenes (**R14**–**R18**) are doubled due to the asymmetry of the monosubstituted disilenes [85], giving rise to two possible directions: the 1-addition pathway, producing the 1-hydroxyadducts, and the 2-addition pathway, producing the 2-hydroxyadducts.

**R14** [85]: H(Me)Si=SiH_2_ + H_2_O
**R15** [85]: HFSi=SiH_2_ + H_2_O
**R16** [85]: H(C≡CH)Si=SiH_2_ + H_2_O
**R17** [85]: H(H_2_N)Si=SiH_2_ + H_2_O
**R18** [85]: H(H_2_N)Si=SiH_2_ + HF

The effect of each substituent is weakly inductive (Me), strongly inductive (F), π-conjugated (C≡CH), and both inductive and π-conjugated (NH_2_). Four initial complexes, two C_N_ and two C_E_, are found in **R14** and **R16**, while only two complexes each are found in **R15, R17**, and **R18**, with two C_N_ in **R15**, one C_N_ and one C_E_ in **R17**, and two C_E_ in **R18**. The paired interacting orbitals clearly indicate whether the initial interaction is electrophilic or nucleophilic [86].

Following the theoretical capture of stable initial complexes at the initial stage of bimolecular reactions, regardless of the polarity of the double bond, by Takahashi et al. [77], the formation of initial or precursor complexes was theoretically reported in various bimolecular reactions involving double-bonded compounds [87,88,89,90,91,92,93,94,95,96,97,98]. Noncovalent interactions of heavy alkenes with H_2_O and HCl were investigated in detail and reported [99]. A mechanism involving initial complexation is consistent with several experimental results, such as a systematic study of the kinetics and mechanisms for five different silenes (ArAr’Si=CH_2_; Ar = Ar’ = 2-MeC_6_H_4_; Ar = Ph, Ar’ = 2,6-Me_2_C_6_H_3_; Ar = Ph, Ar’ = 2,4,6-Me_3_C_6_H_2_; Ar = 2-MeC_6_H_4_, Ar’ = 2,6-Me_2_C_6_H_3_; Ar = Ar’ = 2,6-Me_2_C_6_H_3_) by laser flash photolysis techniques [100], Arrhenius parameters for the reaction of transient silene 1,1-diphenyl-2-neopentylsilene investigated by laser flash photolysis [101], and various distinctive reactions of trisilaallene and 2-germadisilaallene with a variety of reagents, including water, alcohols, acetone, and haloalkanes [102].

Noncovalent VDW forces are very weak and easily disturbed by thermal energy at room temperature. While experimentally detecting stable initial complexes at room temperature is challenging, the efficiency of VDW interactions can be determined spectroscopically. The effect of curvature strain and VDW forces on the interlayer vibrational modes of WS_2_ nanotubes has been reported as a redshift of 2.5 cm^−1^ using confocal micro-Raman spectroscopy [103]. Vibration modes are promising probes for assessing VDW interactions and can be used in various materials, including biological systems. Terahertz vibrations, in particular, are powerful for detecting weak interactions [104,105,106]. Named after van der Waals, who introduced attractive interactions between neutral molecules in his equation of state, VDW interaction is a dispersion interaction of pure quantum physical origin [107,108,109,110,111]. Combined with reliable and well-established density functional theory (DFT) calculations for analyzing vibrational absorption spectra, the effect of dispersive interactions has been directly revealed by observing changes in vibrational spectra at low temperatures where thermal disturbances are suppressed, and VDW interactions become effective against thermal motion [112,113].

#### 2.1.2. Transition States

**Figure 3:**
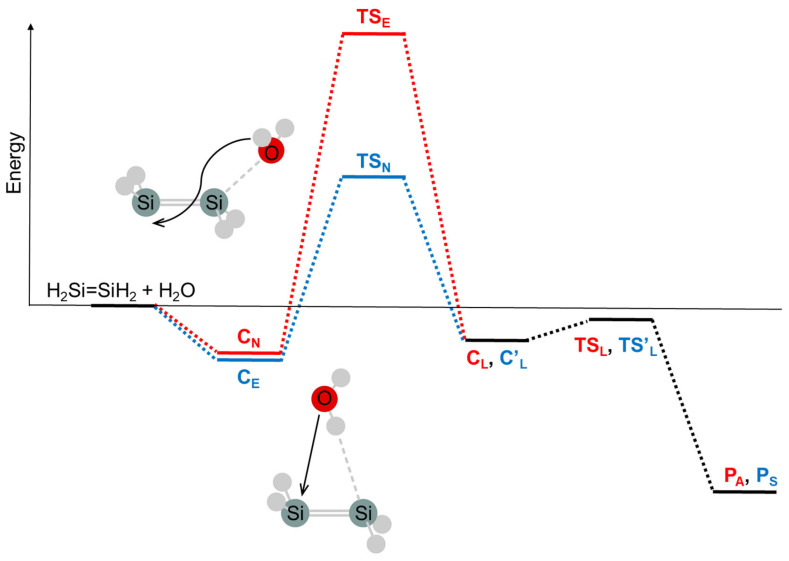
Energy diagram for the water-addition reaction to disilene. C_L_, TS_L_, and P_A_ are the same as C’_L_, TS’_L_, and P_S_, respectively, in the water-addition reaction to disilene, as the two silicon atoms in disilene are not distinguished.

Combining ab initio MO calculations and the FMO theory to search for the transition state offers a good perspective on the mechanism of the reaction of disilene with water, denoted as **R1** in Section 2.1.1 [77,114]. Given that the interaction between disilene and water in the initial complex is very weak, with a stabilization energy of less than 1 kcal/mol, and that the geometry of each reactant is almost unchanged, the energy levels and orbital shapes of the initial complexes are those of the reactants. According to the FMO theory, the orbital interactions between the HOMO and LUMO are crucial. Utilizing the OVGF (outer valence Green’s function) method [115,116,117,118] with a 6-311++G** basis set for the MP2(full)/6-311++G** geometry, the HOMO and LUMO of *trans*-bent disilene are π- and π*-type orbitals, respectively, while the HOMO and LUMO of water are the oxygen lone pair (n) and O-H σ* orbitals, respectively. The formation of the nucleophilic initial complex (C_N_) raises the LUMO (π*) level of disilene and lowers the HOMO (n) level of water. Consequently, the LUMO of C_N_ becomes mainly the LUMO of water (σ*), and the HOMO becomes primarily the HOMO of disilene (π). The secondary step of the reaction after the formation of C_N_ is HOMO (π)–LUMO (σ*) interaction, an electrophilic attack by the water part of C_N_. The hydrogen of water attacks the silicon p_π_ lobe on the other side of the π plane (antarafacial approach), involving rotation around the Si–Si bond, leading to a Lewis adduct (C_L_) via the transition state TS_E_ (Figure 3). From C_L_, the *anti*-adduct (*anti*-silanol) P_A_ is obtained via the four-membered cyclic transition state (TS_L_) discovered by Nagase et al. [119]. On the other hand, the formation of the electrophilic initial complex (C_E_) lowers the HOMO (π) level of disilene and raises the LUMO (σ*) level of water. Thus, the secondary step from C_E_ is the nucleophilic attack of water on disilene through the interaction of the HOMO of water (n) with the LUMO of disilene (π*). Since the n orbital of oxygen is nearly orthogonal to the p_π_ orbital on approach to the Si atom, the sterically favored *syn*-approach is selected, forming the Levis adduct (C_L_’) via transition state TS_N_ and the *syn*-adduct (*syn*-silanol) P_S_ via transition state TS_L_’ (Figure 3).

For large systems, DFT calculations provide a relatively inexpensive alternative to more established quantum-chemical approaches for FMOs. Within the DFT framework, long-range corrected functionals typically yield accurate HOMO and LUMO energies corresponding to ionization potentials and electron affinities, whereas conventional and widely used functionals such as hybrid B3LYP [120] significantly underestimate these orbital energies [121]. FMOs have achieved great success in describing chemical reactivity, particularly for small systems. However, for large systems, the delocalization of canonical molecular orbitals makes it difficult for FMOs to highlight the locality of the chemical reactivity. To obtain localized molecular orbitals that also reflect the frontier nature of chemical processes, the concepts of frontier molecular orbitalets [122] and principal interacting orbital analysis [123] have been recently developed for designing the reactivity of large systems. Additionally, to seamlessly integrate the quantum chemical calculations with chemical intuition, Glendening et al. proposed a practical algorithm for calculating natural bond orbital (NBO)-based resonance natural bond orbitals, which can accurately describe the localized bond shifts in reactive chemical processes [124].

**Figure 4:**
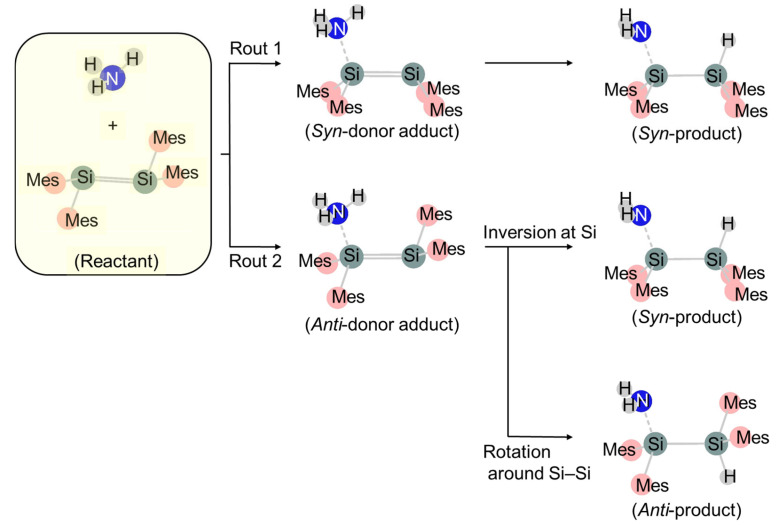
Generalized mechanism for nucleophilic addition of ammonia to disilene.

A generalized mechanism for nucleophilic addition to disilene was recently proposed by McOnie et al. [93] based on computations of the ammonia-addition reaction to tetramesityldisilene. There are two unique approaches for nucleophiles to disilene: approaching from the base of pyramidal Si (Route 1) or approaching from the apex of pyramidal Si (Route 2) (Figure 4). If the nucleophile or the substituents on disilene are small, Route 1 may be followed. This route requires an inversion at Si, followed by an intramolecular transfer of the hydrogen, to provide a *syn*-product, either in two steps or as a concerted reaction. When the substituents are bulky, Route 2 is preferred.

The mechanism of the disilene–water model reaction [77,114], i.e., the formation of an initial complex followed by transition states and intermediate complexes to stereoselectively afford products, has been applied to a variety of bimolecular reactions, such as 1,2-addition to dimetallenes, silene, and trimethylsilylketene [80,83,84,85,86,87,90,91,92,97,125,126,127], abstraction reactions of disilenes and digermenes with haloalkanes [88,89], NH bond activation of ammonia and amines by ditetrelene [93], addition to ring-containing silenes, silabenzenes, germabenzenes, cyclic dimetallaalkenes, and fused tricyclic dimetallenes [94,95,96,128], N-H and O-H bond cleavage catalyzed by single-walled silicon carbide nanotube [98], gold catalyzed cycles [129], the reaction of sulfonyl-containing compounds with ditetrelenes [130], N_2_O activation by substituted disilenes [131], the reaction of disilenes with nitrous oxide [132], and the reactions of phosphino disilenes and their derivatives with an E=E (E = C, Si, Ge, Sn, and Pb) double bond [133]. Alcohols and water [69,134,135,136,137,138,139], as well as amines [140,141,142,143,144,145,146], have been observed to undergo addition reactions with dimer π-bonds on silicon surfaces due to the critical role of reactions at silicon surfaces in disilene chemistry. An investigation of the addition of (2-ethynyl-3-methoxy-2-methylcyclopropyl)benzene to Tip_2_Si=SiTipPh, a disilene with an asymmetric substitution pattern, indicated a stepwise mechanism involving a biradical intermediate based on the regiochemistry of the ring-opened products [147].

#### 2.1.3. Reactivity

The calculations, combined with FMO examinations, indicate that two pathways are viable for the reaction of disilene with water, providing the *syn*- and *anti*-products [77,114]. In the model disilene-water addition reaction, the pathway via TS_N_ is favored over the pathway via TS_E_, as the energy barrier of TS_E_ from C_N_ is higher than that of TS_N_ from C_E_. The preference for the two pathways in the actual reaction of disilene and silene with various alcohols will depend on the electronic and steric effects of the substituents on the disilene and silene, the nature of the nucleophiles and electrophiles, and other factors [80,83,84,85,86,87,88,89,90,91,92,93,94,95,96,97,98,148]. Experimentally, disilenes R*PhSi=SiPhR* (R* = supersilyl = Si*t*Bu_3_) are yellow, water- and air-sensitive crystals that undergo 1,2-addition with H_2_O to convert to disilanes R*PhHSi–SiOHPhR* [149], where *syn*-addition is preferred to *anti*-addition, as predicted theoretically [77]. Several experimental results on 1,2-addition reactions to disilenes have been reported in the literature [150]. Substituent effects on the reactivity of silenes and digermenes have been investigated experimentally and computationally [151,152]. Additionally, the addition reaction of silenes with alcohols depends on the polarity of the Si=C bond [153,154,155].

### 2.2. Unimolecular Rearrangements

**Figure 5:**
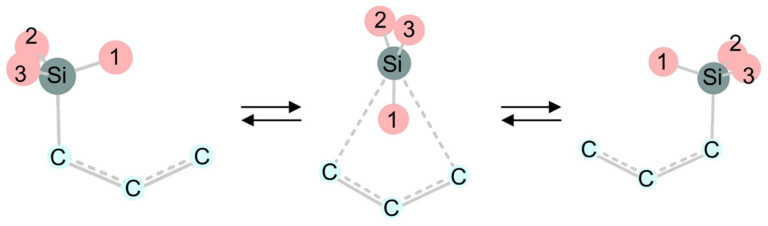
1,3-Silyl migration of allylsilanes with symmetry-allowed suprafacial inversion of configuration at the migrating silicon.

Unimolecular or intramolecular rearrangements in organometallic compounds can be categorized into three different types: those proceeding via the breaking and reforming of σ-bonds (sigmatropic rearrangements), π-bonds (haptotropic rearrangements), and both σ- and π-bonds (dyotropic rearrangements) [156]. In sigmatropic rearrangements, the Woodward–Hoffman (W–H) rule [157] dictates that suprafacial 1,3-migration with retention at the migrating center (suprafacial-retention) is symmetry-forbidden, whereas antarafacial-retention and suprafacial-inversion are allowed, though they are usually sterically disfavored. Thermal 1,3-silyl migration in allylic silanes has been experimentally reported by Kwart et al. to proceed concertedly with a symmetry-allowed suprafacial-inversion of the configuration at the migrating silicon (Figure 5), consistent with the W–H rule [158,159]. This experiment led to the long-held belief that the W–H rule for 1,3-migration was valid for silicon systems as well as carbon systems, at least until the development of high-speed and high-performance supercomputers enabled highly reliable ab initio MO calculations for large-scale systems.

#### 2.2.1. 1,3-Sigmatropic Silyl Migration from Carbon to Carbon Featuring High Coordination

Theoretical studies on 1,3-sigmatropic silyl migration of allylsilanes using ab initio MO calculations were published simultaneously and independently by two Japanese groups [160,161]. Both concluded impressively that the W–H rule is violated in 1,3-sigmatropic silyl migration of allylsilanes. That is, the symmetry-forbidden suprafacial-retention pathway is preferred to the symmetry-allowed suprafacial-inversion pathway. The stereochemical preference different from Kwart et al.’s findings is likely due to the bulky substituents at silicon in their experiments. Subsequently, experimental evidence of retention stereochemistry in the thermal 1,3-sigmatropic silyl migration of allylic silanes was reported, demonstrating that the stereochemical outcome highly depends on the substitutions at silicon [162]. Carbon-to-carbon 1,3-sigmatropic shifts with retention of the configuration at the migrating group have also been reported, such as in GeCl_2_ cycloaddition reactions to unsaturated organic compounds [163] and in the interaction process of deactivated silylenes with buta-1,3-diene [164]. The lowest energy pathway in a computational study of the 1,3-sigmatropic rearrangement of 2-vinylsilirane to silacyclopent-3-ene is a symmetry-allowed suprafacial process with inversion of the configuration at the migrating group [165]. A stepwise mechanism may be the dominant reaction pathway in several theoretically studied particular 1,3-sigmatropic rearrangements [166]. A thermal aromatic 1,3-silyl migration was discovered in unprecedented aryne 1,2,3,5-tetrasubstitution by 3-silylaryne and allyl sulfoxides [167].

**Figure 6:**
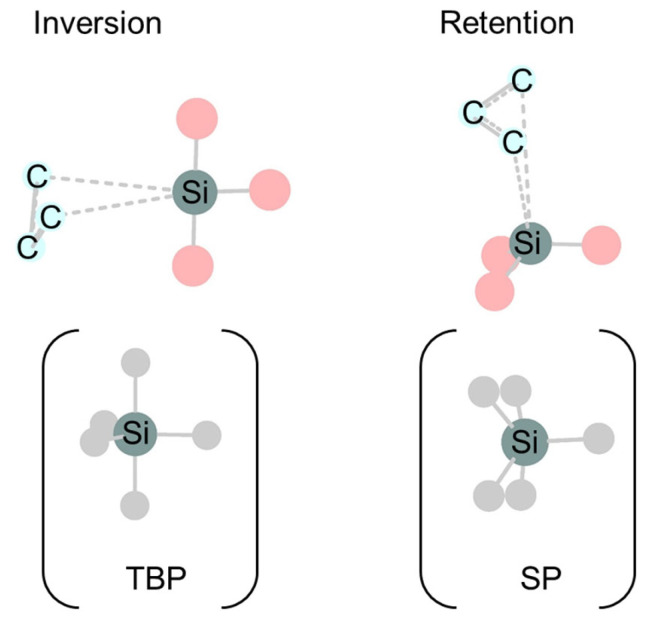
The trigonal bipyramidal (TBP) and square pyramidal (SP) transition structures in 1,3-sigmatropic silyl migration of allylsilanes.

Theoretical studies of 1,2-migration of H, CH_3_, CH=CH_2_, SiH_3_, and GeH_3_ groups on the P and As atoms indicate that the transition states for the migrations of the methyl and vinyl groups, and the hydrogen atom, are high in energy, while the migration of the groups with the heavier elements of Si and Ge utilizes their hypervalency to have a small activation energy [168]. Kwart et al. proposed an orbital diagram for the 1,3-silyl migration of allylsilane, implicitly assuming a rather unusual trigonal bipyramidal (TBP) transition structure, in which two axial positions at the pentacoordinate silicon are occupied by allyl carbons. The pathway via the TBP transition structure is a suprafacial inversion (Figure 6). Alternatively, the migration could proceed with retention of the configuration at silicon by adopting another pentacoordinate silicon structure, a square pyramidal (SP) structure around the silicon (Figure 6). The pathway via the SP transition structure is suprafacial retention. In the calculations, the two suprafacial pathways with different stereochemical configurations at silicon were indeed obtained.

**Figure 7:**
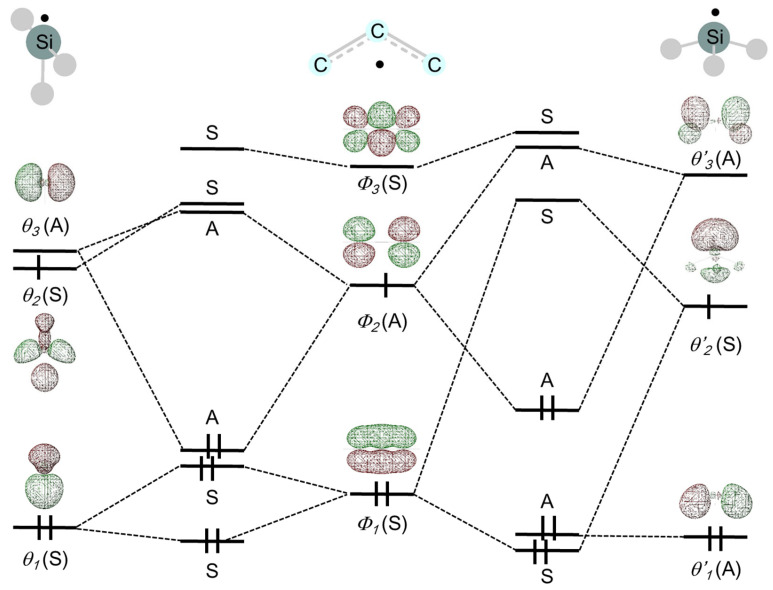
Interaction diagram of frontier orbitals in TBP and SP transition structures for 1,3-silyl migration of allylsilanes. The frontier orbitals of the silyl and allyl radicals interacting in the transition structures are constructed by the three π orbitals (*ϕ*_1_, *ϕ*_2_, and *ϕ*_3_) of the allyl radical and the three 3p orbitals (*θ*_1_, *θ*_2_, and *θ*_3_) or (*θ*’_1_, *θ*’_2_, and *θ*’_3_) of the silyl radical. The symmetry notations S (symmetric) and A (antisymmetric) refer to the planes that bisect the ally CCC plane.

Examining the interactions between silyl and aryl radicals in the transition structures qualitatively reveals the retention preferences (Figure 7). The LUMO, SOMO (singly occupied molecular orbital), and HOMO of the allyl radical are anti-bonding, non-bonding, and bonding orbitals, respectively. The LUMO and SOMO of the silyl radicals involved in the interaction are the same for both the TBP and SP transition structures, i.e., the p-orbitals parallel and perpendicular to the allylic chain, respectively. The HOMO of the silyl radical differs between the TBP and SP transition structures, being the p-orbital perpendicular and parallel to the allyl radical chain, respectively. The major stabilization of the TBP transition structure for suprafacial-inversion is caused by the interaction between the low-lying LUMO of the silyl radical with the non-bonding SOMO of the allyl radical, as predicted by the W–H rule. On the other hand, due to the difference in HOMO, in case of the SP transition structure for suprafacial-retention, in addition to the stabilization by the interaction of the low-lying LUMO of the silyl radical with the non-bonding SOMO of the allyl radical, there is also stabilization by the interaction of the subjacent bonding orbital of the allyl radical with the SOMO of the silyl radical.

#### 2.2.2. 1,3-Silyl Migration Between Heteroatoms

It has been established that intramolecular thermal 1,3-sigmatropic silyl migration occurs concertedly from carbon to carbon in allylic silanes [158,159] and between heteroatoms in silylmethyl ketones [169] with optical active silyl groups. The latter migration in silylmethyl ketones is known as the Brook rearrangement. These migrations were considered to be mechanically distinguished by a striking difference in stereochemical outcome. That is, 1,3-silyl migration in allylic silanes occurs with inversion stereochemistry, whereas in silylmethyl ketones it occurs with retention stereochemistry. However, theoretical studies by Takahashi et al. [160] and Yamabe et al. [161] revealed, as mentioned in Section 2.2.1, that two concerted pathways leading to retention and inversion in silicon are both allowed for thermal 1,3-silyl migration in allylsilanes, with the retention pathway being lower in energy. These reports on retention preference in the thermal 1,3-silyl migration of allylsilane stimulated further the theoretical investigations into the mechanism of 1,3-silyl migration in silylmethyl ketones.

In the theoretical study of concerted 1,3-silyl migration in formylmethylsilane, the calculated activation energy, as well as retention stereochemistry, are in good agreement with experimental results for silylmethyl ketones [170]. 1,3-Silyl migration in both allylsilane and formylmethylsilane proceeds concertedly, without the formation of intermediates, via a four-membered cyclic transition structure, with retention stereochemistry around the silicon. However, there are essential differences in the transition structures. 1.3-Silyl migration with retention in formylmethylsilane is best explained as an intramolecular nucleophilic substitution at silicon, whereas the corresponding migration in allylsilanes is characterized as an electrocyclic sigmatropic rearrangement controlled by subjacent orbital interactions [171,172]. Reaction pathways involving 1,3-silyl migration between heteroatoms have been reported computationally and experimentally in various reactions [173,174,175,176,177,178,179,180,181,182,183,184,185,186]. In the benzidine rearrangement, an unprecedented suprafacial symmetry-allowed 1,3-sigmatropic shift from nitrogen to carbon with an inversion of the configuration at the migrating nitrogen atom was supported by a systematic investigation of experiments and theoretical calculations [187].

## 3. Photochemical Reactions

The complete active space self-consistent field (CASSCF) method is one of the most appropriate ab initio methods for investigating the reaction pathways on excited states. Since Bernardi et al. reported on the photochemical sigmatropic rearrangement of but-1-ene [188], several photochemical reactions have been extensively studied for carbon systems using the CASSCF method [189,190,191,192,193,194,195,196,197,198]. It is commonly understood that photochemical investigations using the CASSCF method require a careful selection of the correct orbitals for active space. With adequately selected orbitals for the active space and a sufficiently large basis set, the excitation energies calculated at the MP2-CAS level are in excellent agreement with the experimentally observed UV absorption maxima [199]. In addition to the ones presented here, theoretical studies on the photochemical reactions of organosilicon compounds using the CASSCF method have been published [200,201,202].

Full photodynamics calculations are prohibitively expensive for all but the smallest molecules, unless simplifying approximations are employed. Time-dependent DFT (TDDFT) offers a promising and relatively inexpensive alternative to more accurate, but costly, quantum chemical approaches, for on-the-fly calculations of excitation energies [203,204]. Several applications of TDDFT to photochemical reactions in silicon chemistry are documented in the literature, primarily focusing on the investigation of solid-state materials such as crystals, surfaces, two-dimensional materials, and nanomaterials [205,206,207,208,209].

### 3.1. Interconversion

**Figure 8:**
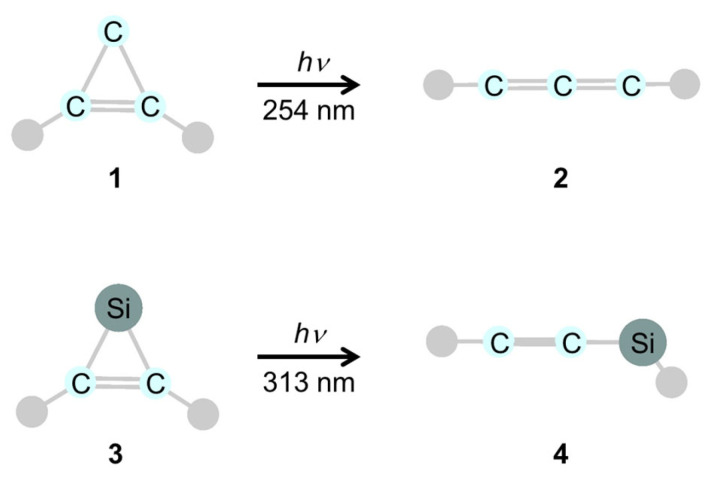
Photochemical reactions of cyclopropenylidene (**1**) and silacyclopropenylidene (**3**).

Cyclopropenylidene (**1**, c-C_3_H_2_) is abundant in molecular clouds of interstellar space and plays a decisive role in the chemistry of interstellar clouds [210,211,212,213]. The silicon analog, silacyclopropenylidene (**3**, c-C_2_H_2_Si), also appears to be of interest, especially in astrochemistry and structural chemistry [214]. Maier et al. reported the synthesis and photoisomerization of **1** and **3** [215,216]. It was shown that the photochemical interconversion of **1** occurs between three C_3_H_2_ isomers: **1**, propargylene (**2**), and vinylidenecarbene [215]. On the other hand, the photochemical reaction of **3** is more complex, with several isomers detected during the reaction [216]. The reaction products detected by infrared spectroscopy are **2** and ethynylsilylene (**4**), upon irradiation with 254 nm light [215] for **1** and 313 nm light for **3** [216], respectively (Figure 8). Recently, crossed beam experiments and computational studies have revealed a synthetic route to singlet **4** (HCCSiH; X^1^A′) [217]. The ground-state structures, energetics, and vibrational frequencies observed in photochemical reactions of **1**, **3**, the germanium analog (GeC_2_H_2_), SiC_4_H_2_ isomers, and Si_2_C_5_H_2_ isomers have been intensively investigated by theoretical chemists [218,219,220,221,222,223,224,225,226,227,228].

The photochemical reaction pathway of **3** has been investigated in detail by Takahashi et al. using the CASSCF method [199]. The excited state through which the photochemical reaction proceeds was carefully chosen because the forbidden state ^1^(σ_SiC_ ⟶ 3p) is energetically close to the allowed state, ^1^(n ⟶ 3p). For examining the orbitals and planning the active space, the natural orbitals are examined. Then, one π_CC_ and two σ_SiC_ orbitals, and one lone-pair orbital at silicon are selected. The use of the eight active orbitals to describe the present system is justified as follows: The photochemical reaction treated here is the Si–C bond cleavage. The first allowed band is best described as n ⟶ 3p(Si). The carbon π orbital (π_CC_) is mixed in an antibonding manner with the 3p(Si) orbital. The photochemical reaction from the S_1_ ^1^(n ⟶ 3p) excited state of *c*-C_2_H_2_Si leads to an S_1_/S_0_ conical intersection (CI), and the photoexcited state decays nonradiatively to S_0_. Relaxation on the S_1_ surface leads to the cleavage of the C–Si single bond, followed by hydrogen migration from carbon to silicon with ring opening in the dark reaction.

### 3.2. Sigmatropic Rearrangement

Photochemical 1,3-silyl migration is of particular interest from a mechanistic perspective. Numerous reactions involving photochemical silyl migration from carbon to carbon [229,230] or between heteroatoms [231,232,233,234,235,236,237,238,239,240,241,242,243,244,245] have been reported in the literature. However, the stereochemical and mechanistic details have rarely been described [230]. The stereochemistry of 1,3-sigmatropic rearrangements is systematically explained by the well-established Woodward–Hoffmann (W–H) rules based on orbital symmetry [157]. In photochemical 1,3-sigmatropic rearrangements, the migrating group moves according to a supra process and retains its configuration, whereas thermal suprafacial rearrangements occur with inversion of configuration at the migrating center (Figure 5). While the W–H rule correctly predicts the stereochemistry in reactions of organic compounds with a carbon framework, it is of great interest to investigate whether this rule can be applied to the stereoselection of organosilicon compounds. According to modern experiments and quantum chemical calculations, low-lying crossings on the potential energy surface (conical intersection: CI) are a common feature of excited states relevant for photochemical reactions.

An experimental study of the photochemical 1,3-silyl migration in allylsilanes was reported by Kira et al. in 1989 [230]. The photochemical migration occurs with inversion of the silyl configuration, contrary to the W–H rules. The configuration of the photochemical products was identified by performing a thermal reverse reaction that occurs with inversion, according to Kwart et al. [158,159]. On the other hand, the thermal 1,3-silyl migration of allylic silanes with inversion of the configuration was doubted by Brook [246]. Furthermore, theoretical studies have shown that the thermal 1,3-silyl migration of allylsilanes favors retention of configuration [160,161], and experiments with stereochemically rigid 4-*tert*-butylsilacyclohaxane derivatives support the preference for retention [162].

The photochemical 1,3-silyl rearrangement of allylsilanes has been theoretically investigated by Takahashi [247], focusing on stereochemistry and reaction mechanism. To select the correct orbitals for the active space in the photochemical investigation using the CASSCF methods, the natural orbitals are examined, and the six active orbitals are selected: the π-, π*-, σ-, and σ*-orbitals in the C=C bond and the σ- and σ*-orbitals in the Si–C bond that is cleaved. In the system with a complete active space comprising six electrons in six orbitals, denoted by CAS(6,6), two electrons among six are fully coupled in a σ_CC_ bond, leaving two possible spin couplings for the remaining four electrons. The driving force that controls the generation of ground-state photoproducts is expected to be provided by recoupling these four electrons in the partly fragmented bonds that occur when the system decays. The use of six active orbitals is justified as follows: For the description of the photochemical 1,3-sigmatropic shifts in a carbon system, four electrons in four orbitals, denoted by CAS(4,4), is the usual active space, as reported by Bernardi et al. [188]. However, better Si–C bond lengths, related to the possibility of the dissociation of an allyl group, are given in CAS(6,6) than in CAS(4,4).

**Figure 9:**
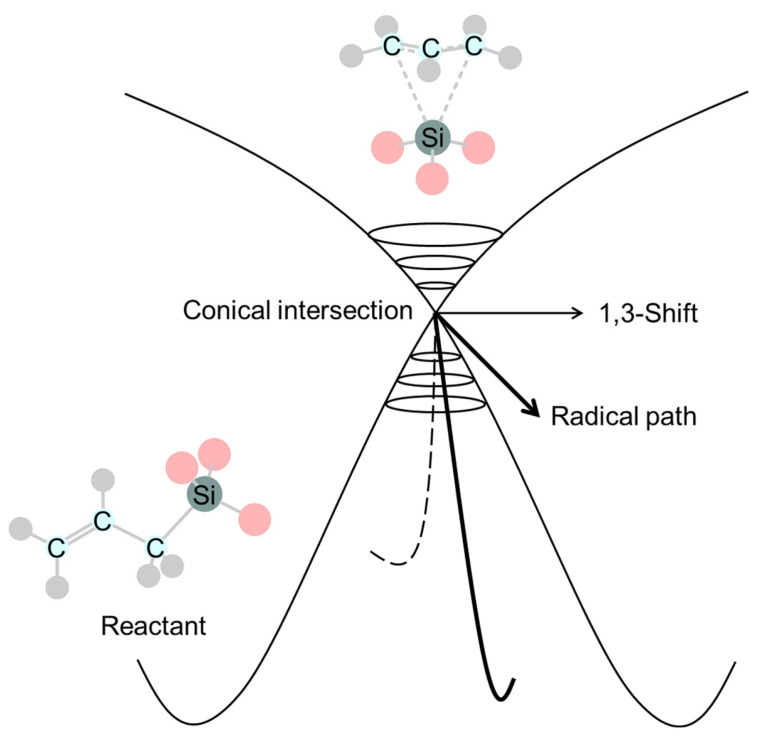
A characteristic conical intersection (CI) structure common to photochemical sigmatropic shifts reported as a ubiquitous control element [194].

The stereochemistry of the photochemical 1,3-silyl rearrangement of allylsilanes was theoretically confirmed to be retained, in agreement with the W–H rule [247]. The calculated CI structure is consistent with ubiquitous control elements (Figure 9) in photochemical sigmatropic rearrangements within the carbon framework [194], indicating the possibility of a dissociation pathway to radicals, in addition to the 1,3-shift pathway. Fifteen years after Takahashi ‘s theoretical work [247], Hammer et al. reported experimentally a stereoselective photochemical 1,3-sigmatropic silyl shift and the existence of a silyl/allyl CIs [248].

## 4. Conclusions

In this review article, we introduce the theoretical studies on the thermal and photochemical reactions of organosilicon compounds, focusing on our work conducted in 1997–2005. We also comprehensively introduce the related experimental and theoretical studies by previous and recent researchers. In the late 1980s, thermal reactions of unsaturated silicon compounds could be treated with quantitative accuracy using ab initio MO calculations without experimental parameters, allowing for the theoretical prediction of reaction path preferences. Furthermore, the development of high-performance and high-speed supercomputers has accelerated the ab initio MO calculations of larger molecules, containing heavier elements such as silicon. In particular, a frequency analysis to distinguish between the transition states and local minima, which are essential for investigating reaction pathways, required time-consuming, large-scale calculations. Theoretical studies of photochemical reactions started a little bit later and still continue to intrigue theoretical chemists, with recent advances in experimental techniques to capture photochemical reactions.

## Data Availability

No new data were created or analyzed in this study. Data sharing is not applicable to this article.
